# Supplementary material for: Development of a modular stress management platform (Performance Edge VR) and a pilot efficacy trial of a bio-feedback enhanced training module for controlled breathing
Source: PLoS One. 2021 Feb 2;16(2):e0245068. doi: 10.1371/journal.pone.0245068 (PMC7853514; doi:10.1371/journal.pone.0245068)
Supplement: S1 File — (DOCX) [file pone.0245068.s001.docx]

# Development of a modular stress management platform (Performance Edge VR) and a pilot efficacy trial of a bio-feedback enhanced training module for controlled breathing

Murielle G. Kluge^1,2+^, Steven Maltby^1,2+^, Nicole Walker^3^, Neanne Bennett^4^, Eugene Aidman^2,5,6^, Eugene Nalivaiko^1,2#^ & Frederick Rohan Walker^1,2#*^

^1^Centre for Advanced Training Systems, Faculty of Health & Medicine, The University of Newcastle, Callaghan NSW 2308, Australia.

^2^School of Biomedical Sciences & Pharmacy, Faculty of Health & Medicine, The University of Newcastle, Callaghan NSW 2308, Australia.

^3^Army School of Health.

^4^Department of Defence

^5^Land Division, Defence Science & Technology Group, Edinburgh, SA, Australia.

^6^School of Psychology, The University of Sydney, Sydney, NSW, Australia.

***Corresponding Author:**

Email: [rohan.walker@newcastle.edu.au](mailto:rohan.walker@newcastle.edu.au) (FRW)

# Supporting Information:

**Supplementary Table I:** **Pre-Training Questionnaire.** Administered before training on sessions 1 – 3.

**Supplementary Table II:** **Post-Training Questionnaire – Hardware & Software Feedback.** Administered after training session 1.

**Supplementary Table III: Post-Training Questionnaire – Skills Feedback.** Administered after training session 3.

# Supplementary Tables:

**Supplementary Table I: Pre-Training Questionnaire.** Administered before training on sessions 1 – 3.

| **Question** |
| --- |
| Please specify your age |
| How would you rate your level of attention to your breathing on an everyday basis before participating in this study? [No / Some / Frequent] |
| [Session 1 only] Have you engaged in controlled breathing before participating in this study? (Controlled breathing is defined as the performance of an intentional slow and steady breath, such as during meditation) [Yes/No] |
| How often do you engage in any type of controlled breathing activity? |
| [Session 2 & 3] Since the initial VR training session how would you rate changes in level of attention to your breathing? [Less / No change / More] |
| [Session 2 & 3] Have you used the controlled breathing skill at any time after the experiment? [Yes / No] |
| [Session 2 & 3] How frequently have you used the skill since you left the experiment? |

**Supplementary Table II: Post-Training Questionnaire – Hardware & Software Feedback.** Administered after training session 1.

| **Question** |
| --- |
| How would you rate the overall level of comfort whilst wearing the VR equipment (headset, biometric belt, hand-held controllers and/or audio equipment)? |
| How would you rate the level of comfort wearing the VR headset? |
| How would you rate the level of comfort wearing the biometric belt? |
| How would you rate the level of comfort wearing the headphones? |
| How would you rate the level of comfort holding the controllers? |
| Did any of the hardware components impede your ability to move freely or participate in the training? [If yes, please provide more information] |
| How do you rate the overall VR experience, including both educational and exercise components? |
| Is there anything you would change about the overall VR experience? |
| Rate the ease of navigation through the module components and between exercises. |
| Rate how intuitive you found the VR interface (i.e. how you interact with the VR) through the module. |
| If you have any additional comments on the VR interface, please provide details |
| How easy did you find it to understand and follow the educational components of the module? |
| Rate the explanations and presenter, in relation to the clarify and flow of content |
| Rate the suitability of the level of complexity of the educational content |
| Rate the complexity of the educational content |
| How well did the graphics accompanying educational content help you understand the concepts? |
| How well did the appearance, speech and demeanour of the presenter align with the concepts being taught? |
| After using the tool, did you learn something new about the use, benefit and appropriate engagement with controlled breathing? |
| Provide general feedback and comments on the educational component of the module |
| What did you like about the educational component? |
| What did you dislike about the educational component? |
| Is there anything you would change about the educational component? |
| How well did you understand the instructions given for the exercises in the module? |
| How difficult did you find the general tasks presented in the module? |
| How difficult did you find the final crossbow shooting exercise specifically? |
| How appropriate did you think the duration of the breathing exercises was? |
| Rate the overall appearance of the exercise components of the module, including design, graphics, images and overall environment. |
| Please provide more information on aspects of the appearance that you liked / disliked. |
| How well did the graphics support your engagement with the VR module? |
| How helpful was the biofeedback function (visualisation of your breathing pattern and rate)? |
| What did you like about the exercise component? |
| What did you dislike about the exercise component? |
| Is there anything you would change about the exercise component of the VR module? |
| To what extent do you agree with the statement “This is a useful tool to develop and practice controlled breathing”? |
| After using the tool once, that you now have a better understanding of the concept of controlled breathing? |
| After using the tool once, do you have a better understanding of your breathing and how it changes in different conditions? |
| After using the tool once, are you more competent in controlling your own breathing? [If no, why not?] |
| Based on your experience with this training tool, do you think that you might engage in controlled breathing the next time you experience a stressful event? [If no, why not?] |

**Supplementary Table III: Post-Training Questionnaire – Skills Feedback.** Administered after training session 3.

| **Question** |
| --- |
| How do you rate the overall VR experience of this module, including both the educational and exercise components after using it multiple times? |
| Is there anything you would change about the overall VR experience? |
| Please rate the ease of navigation throughout the module components and between exercises. |
| Did using the tool on multiple occasions change the way you feel about navigation and user interface? [If yes, how?] |
| Did the exercises become easier after using the tool multiple times? |
| How appropriate did you think the duration of the breathing exercises was? |
| Did using the tool on multiple occasions change the way you feel about the navigation and user interface? |
| How helpful was the biofeedback function in this module (the visualisation of your breathing pattern and rate)? |
| To what extent do you agree with the statement that “This is a useful tool to develop and practice controlled breathing”? |
| Do you think that after using this tool multiple times you now have a better understanding of your breathing and how it changes in different conditions than you did after using it only once? |
| Do you think after using the tool multiple times that you are now more competent in controlled your own breathing than you were before? [If no, why not?] |
| Do you think, based on this training tool, that you might engage in controlled breathing the next time you experience a stressful event? [If no, why not?] |
