# Supplementary material for: Development of a modular stress management platform (Performance Edge VR) and a pilot efficacy trial of a bio-feedback enhanced training module for controlled breathing
Source: PLoS One. 2021 Feb 2;16(2):e0245068. doi: 10.1371/journal.pone.0245068 (PMC7853514; doi:10.1371/journal.pone.0245068)
Supplement: Attachment — Submitted filename: REBUTTAL Performance Edge VR Rev1.docx [file pone.0245068.s002.docx]

**Professor Rohan Walker**

Assistant Dean of Partnerships and Innovation

School of Biomedical Sciences and Pharmacy

Faculty of Health and Medicine

University of Newcastle

Callaghan, NSW Australia 2308

T: +61 2 4921 5012

F: +61 2 4921 5141

[rohan.walker@newcastle.edu.au](mailto:rohan.walker@newcastle.edu.au)

<https://www.newcastle.edu.au/profile/rohan-walker>

**16 December 2020**

**Dear Editor in Chief Joerg Heber,**

We thank the editors and reviewers for their constructive and insightful feedback and the opportunity to resubmit our revised manuscript. We have updated our manuscript entitled **“Development of a modular stress management platform (Performance Edge VR) and a pilot efficacy trial of a bio-feedback enhanced training module for controlled breathing**’.

We have incorporated changes to the manuscript document and figures, as indicated in a detailed point-by-point response to reviewers comments.

As requested, we have updated the Competing Interests statement (pg. 21) as follows: *“All authors were involved in the development of the Performance Edge VR application. Intellectual property relating to the application is owned by The University of Newcastle. The Australian Defence Innovation Hub provided funding for application development and retains rights for its future use. This does not alter our adherence to PLoS ONE policies on sharing data and materials”.* Please update this statement in the online submission portal on our behalf.

Thank you for your consideration of our work, which we believe will be of interest to *PLoS ONE* readers. We hope that you will now consider it suitable for publication in *PLoS ONE.*

Kind regards,

Prof. Rohan Walker

**DETAILED POINT-BY-POINT RESPONSE TO COMMENTS:**

***Editorial Office Feedback:***

*1. Please ensure that your manuscript meets PLOS ONE's style requirements, including those for file naming. The PLOS ONE style templates can be found at* [*https://journals.plos.org/plosone/s/file?id=wjVg/PLOSOne_formatting_sample_main_body.pdf*](https://journals.plos.org/plosone/s/file?id=wjVg/PLOSOne_formatting_sample_main_body.pdf) *and* [*https://journals.plos.org/plosone/s/file?id=ba62/PLOSOne_formatting_sample_title_authors_affiliations.pdf*](https://journals.plos.org/plosone/s/file?id=ba62/PLOSOne_formatting_sample_title_authors_affiliations.pdf)

**Article formatting has been updated to comply with PLOS ONE style requirements and guidelines.**

*2. We note that you have included the phrase “data not shown” in your manuscript. Unfortunately, this does not meet our data sharing requirements. PLOS does not permit references to inaccessible data. We require that authors provide all relevant data within the paper, Supporting Information files, or in an acceptable, public repository. Please add a citation to support this phrase or upload the data that corresponds with these findings to a stable repository (such as Figshare or Dryad) and provide and URLs, DOIs, or accession numbers that may be used to access these data. Or, if the data are not a core part of the research being presented in your study, we ask that you remove the phrase that refers to these data.*

**The indicated sentence referred to measurements of baseline unprompted breathing rates at the beginning of each training session. This sentence has been removed from the manuscript, as the point is not critical to the key study findings.**

*3. Thank you for stating the following in the Competing Interests section:*

*[All authors were involved in the development of the Performance Edge VR application. Intellectual property relating to the application is owned by The University of Newcastle. The Australian Defence Innovation Hub provided funding for application development and retains rights for its future use.].*

*Please confirm that this does not alter your adherence to all PLOS ONE policies on sharing data and materials, by including the following statement: "This does not alter our adherence to PLOS ONE policies on sharing data and materials.” (as detailed online in our guide for authors* [*http://journals.plos.org/plosone/s/competing-interests*](about:blank)*).  If there are restrictions on sharing of data and/or materials, please state these. Please note that we cannot proceed with consideration of your article until this information has been declared.*

*Please include your updated Competing Interests statement in your cover letter; we will change the online submission form on your behalf.*

*Please know it is PLOS ONE policy for corresponding authors to declare, on behalf of all authors, all potential competing interests for the purposes of transparency. PLOS defines a competing interest as anything that interferes with, or could reasonably be perceived as interfering with, the full and objective presentation, peer review, editorial decision-making, or publication of research or non-research articles submitted to one of the journals. Competing interests can be financial or non-financial, professional, or personal. Competing interests can arise in relationship to an organization or another person. Please follow this link to our website for more details on competing interests:* [*http://journals.plos.org/plosone/s/competing-interests*](about:blank)

**The Competing Interests statement has been updated (pg. 21). The updated statement is also included in the cover letter, as requested.**

*4. In your Methods section, please provide additional information about the participant recruitment method and the demographic details of your participants. Please ensure you have provided sufficient details to replicate the analyses such as:
a) the recruitment date range (month and year),
b) a description of any inclusion/exclusion criteria that were applied to participant recruitment,
c) a table of relevant demographic details,
d) a statement as to whether your sample can be considered representative of a larger population.*

**We have included additional detail on the study recruitment dates and inclusion / exclusion criteria and representativeness of the study population in the Methods (pg. 7/8). Relevant demographic details are included in Table I.**

*5. Please include additional information regarding the post-training questionnaires used in the study and ensure that you have provided sufficient details that others could replicate the analyses. For instance, if you developed the questionnaire(s) as part of this study and it is not under a copyright more restrictive than CC-BY, please include a copy, in both the original language and English, as Supporting Information.*

**Questionnaires were developed for the current study and have now been included in Supporting Information, as noted in the Methods (pg. 9)**

*6. For papers that describe new methods or tools, these reports must meet the criteria of utility, validation, and availability, which are described in detail at* [*http://journals.plos.org/plosone/s/submission-guidelines#loc-methods-software-databases-and-tools*](http://journals.plos.org/plosone/s/submission-guidelines#loc-methods-software-databases-and-tools)*. Please describe how another researcher could access the Performance Edge VR training module, or provide a link to your code.*

**We have added the following statement in the Methods section (pg. 6/7) – “Intellectual property relating to Performance Edge VR is owned by The University of Newcastle. If interested in accessing Performance Edge VR application for research purposes, please contact the corresponding author.”**

***Review Comments to the Author***:
**Reviewer #1:**

*The manuscript is very well-written and presented, however, I do have few comments/suggestions that may improve the readability.*

**We thank the reviewer for their positive and constructive feedback.**

*Intro/Methods: Please also provide some relevant literature on importance of breathing variability for stress management before directly introducing it in Methods as one of the things you assessed from the VR module.*

**A sentence has been added to the introduction clarifying that controlled breathing consists of deep, slow and steady breathing which can be measured as a reduction in both respiratory rate and variability (p.4). This is outlined in references 21, 22.**

*Overall, I like the discussion where you compare with (many) other similar platforms/devices out there trying to make use of breathing/HR/HRV for biofeedback for stress management, however, I think some of that could be moved up to the Introduction / methods to help make the case as why you chose to do the way you chose to do it.*

**As suggested, we have included a brief summary of existing VR applications integrating biofeedback in the Introduction (pg. 5).**

*Overall, it seems there is over abundance of re-emphasizing as how the design went through multiple iterations and got approval from so and so, but I think some of that is internal to your logistics and unnecessary distraction. It will be great to have more focus on technical aspects of the design. Remember although PLoS one covers quite broad topics, but it is not a typical HCI journal.*

**Efforts have been made to streamline the indicated Methods & Results sections, where we describe the scoping, approval and development processes. The remaining content related to review and approval was deemed relevant to highlight the efforts made to ensure that Performance Edge VR is fit-for-purpose and suited to the intended training environment, and that it has been supported at the highest organisational level of the Australian Defence Forces.**

*2.7 Study protocol: I think it will help to provide more technical details about the type/order/cutoff of filter being used. Did you use all 3 min. of data, or removed first 30-45 sec (I think the manual for Zephyr Bioharness suggest that it may take up to 45 sec to reach a stable level after start of any condition). I think tracking depth of breadth is very important and if the raw data has been collected w/o real-time digital filtering, then it should be discussed, even if authors do not currently use it to provide feedback (but can comment why this may not also be used). I think for a within-subject design, it may be okay to use this metric even after low pass filtering, to help explain if they are engaging in more deeper breaths. Were subjects told to do diaphragmatic breathing or no such instructions were given, if not, why that was not deemed necessary for a breathing intervention? Could it not make some people who may have bias about breathing control from linking it to meditation/yoga to not try to think of this like that?*

**Thank you for this comment. We have added more technical details to the methods section (p. 8). The Equivital life monitor belt used in this study provides real-time data capture and does not have a time delay as suggested for the Zephyr Bioharness. Pre- and post-training breathing data was collected for 3-4min, whilst 3 consecutive minutes of the recording was analysed. A digital Triangular (Bartlett) Smoothing window with window width of 205 samples was used to filter real-time raw data, necessary to remove artefacts and instrumental noise. This has now been noted in the manuscript Methods (pg. 8) Whilst we agree with** **the reviewer’s comment stating that breathing depth is a relevant component of breath control, no analysis was performed due to effects of smoothing on this output. Instead, we focused on breathing rate and consistency as valid measures of breath control as they were not affected by data processing.**

**Participants were instructed to take deep, slow and steady breaths at their personal lowest comfortable breathing rate with no specific reference to diaphragmatic breathing or any other breathing technique. We prioritised easy to follow instructions over a specific rhythm or technique with the learning goal focused on the development of breath awareness for intentional and controlled breathing. This decision is supported by existing literature, which indicates that many different breathing techniques can be effective in the context of stress management, provided they include intentional and rhythmical breathing at a reduced breathing rate (Discussion; p.17-18, line 519-559). Increased breathing depth is a feature which often accompanies intentional, rhythmical breathing at a reduced rate but was not used for biofeedback within our VR application or a primary outcome of this study due to the above-mentioned filtering of the real-time data, which was required for the live biofeedback tool.**

*Section 3.2 - Please provide a higher resolution snapshot in Fig. 2. It is not legible (else I do not see any point of providing it)*

**A higher resolution image has been uploaded for Figure 2.**

*When subject was given bio-feedback, was that of the BR in real-time, or average of last few seconds (if not, why should some sort of moving average filter not be considered?)*

**Users were provided with a real-time respiratory bio-feedback trace and a moving average breathing rate, when exercises integrated live biofeedback. In other exercises, users were provided with their average breathing rate over a 2-minute timeframe and a breathing trace, after completing the exercise. This information is represented in Figure 2 and has been clarified in the figure legend (pg. 10).**

*I see Fig. 2D says 4 bpm that is really low to be considered smooth/continuous/repeatable/sustainable breathing. Please discuss implications for such very low breathing (and thus low O2?).*

**The indicated figure was provided to visualise the graphic design and user interface for Performance Edge VR. The respiratory data included in the figures shows the respiratory trace and rate of one of the research team members, and was not intended to be representative of usage data. An updated Figure 2 has now been included.**

**We note that users were prompted to reduce their breathing rates to their personal “lowest comfortable breathing rate”. The lowest average breathing rate recorded for the study cohort after training session 3 was approximately 4.9 BPM (Figure 6A). No measurements were taken to assess blood oxygenation levels. We do note that literature suggests breathing rates as low as 3 BPM can be performed by experts without effecting oxygen levels (due to associated increases in depth of breathing).**

*Similarly what are the three numbers 20, 8 and 34 in Fig. 2C?*

**The screenshot displays the user’s average breathing rate at baseline (“Normal”; top), after completing 10 deep breaths (middle) and after mild physical activity (standing & sitting 10 times; bottom) to familiarise the user with the physiological changes in their breathing rate. A higher resolution screenshot has been included and the figure legend has been modified to clarify this point (Figure 2; pg. 10).**

*Were subjects told to have some target breathing? If so, how was that target determined or the trajectory that they were supposed to follow? How were they scored / performance assessed?*

**A common feature across effective breathing approaches is intentional and rhythmical breathing at a reduced breathing rate. In alignment with this, Performance Edge VR does not impose a specific target value but rather provides training to maintain a slow and steady breath at a personal user-defined “lowest comfortable breathing rate”. Biofeedback is used in Performance Edge VR as an educational training feature with 3 distinct goals: 1) Visualisation of changes in personal breathing pattern and rates for different activities (Figure 2C), 2) Visualisation of real-time breathing trace to support manual control over breathing and assist with establishing a lowest comfortable breathing rate (depicted in Figure 2B) and as a 3) Performance measure of the trainees ability to maintain their personal lowest comfortable breathing rate in subsequent exercises (without the assistance of real-time biofeedback and with accompanying distractions). Feedback in each exercise was reported to users in comparison to their own personal lowest comfortable breathing rate (ie. faster, same or slower). This was presented after each exercise to provide feedback to users (Figure 2D). Figure 2, the accompanying figure legend and manuscript text (p. 11/12) have been modified to clarify these points. Of note, in the final exercise users perform a secondary shooting task whilst maintaining their lowest comfortable breathing rate. In this case, “performance” feedback included details on both maintenance of breathing rate AND shooting game outcomes (ie. total time, number of arrows used and points to provide some gamification of relative performance).**

*3.4 How long time interval was used to estimate 'lowest comfortable breathing rate'?*

**Within the Performance Edge VR application, the lowest comfortable breathing rate was measured over a 2-minute period during exercise 2. This measurement is used to provide user feedback for comparison in subsequent exercises, as described in detail above. In the research trial protocol, controlled breathing was assessed both pre- and post-training during a 3-minute time period (Figure 1). In both circumstances, users were prompted to “take slow and steady breaths at their lowest comfortable breathing rate”. This is indicated in the Methods (pg. 8).**

*pg 12 top line - where you say at first they used the live respiratory trace as a visual guide - I wonder how do they know if it is not correct or what is that they should try to do to change it to what?
4 lines down when you say performance is provided to the user, please provide more information as how they were scored?*

**Clarification has been integrated into the manuscripts as outlined in the responses to reviewer comments above.**

*Please provide error bars in Figs 3 and 4 and not just averages across 30 subjects for each of the categories.*

**Figures 3 & 4 have been updated to include errors bars or individual user data points, as requested.**

*It was also not clear if some stressors can be integrated in this platform to test this training module for its efficacy as a stress management tool per se.*

**Thank you for this suggestion. Indeed, we intend to add stressors in future expansions of Performance Edge VR and we agree that this will be a useful application of the technology. This has now been noted in the manuscript Discussion (pg. 20).**

**Reviewer #2:**

*“In fact, there is now growing recognition that optimal performance in challenging situations (and importantly during post-exposure recovery), is determined by a positive interaction between external and internal skills and knowledge (9).”
9. Zheng C, Kashi K, Fan D, Molineux J, Ee MS. Impact of individual coping strategies and organisational work–life balance programmes on Australian employee well-being. The International Journal of Human Resource Management. 2016;27(5):501-26.
This does not really seem enough to say “there is growing recognition…”*

**The indicated sentence has been removed.**

*Please fix “‘inoculuate”*

**Typo corrected (pg. 5).**

*Figure 2 should be better quality in the final version because when I zoom on it I can’t really see what’s written*.

**An updated high resolution figure has been uploaded.**

*In "3.1 Identification of a need for new training approaches to support stress management training in Defence"
These are supposed to be results. I’d like to see something showing a result, not just a sentence saying “it is necessary”*

**The content identified has now been moved into the Methods section (pg. 6) to provide context for project initiation. Specific qualitative / quantitative data was not collected for this aspect of the study.**

*“Several potential participants opted not to participate after reading the participant information statement. “
I wonder how many, and whether the sample of people who wanted to do this is somehow biased to high acceptance.*

**We have updated the text to clarify that 3 potential participants opted not to participate in the study after reading the participant information document, due to the length of the study and requirement to attend 3 training visits (pg. 12). We do not believe this factor is likely to have biased the findings or affected the application to the broader population.**

*“Half of all participants reported previous experience with controlled breathing at the beginning of session 1” Where these analyzed separate from others? This should be done to make sure previous experience is not biasing the results.*

**Thank you for this suggestion and great point. We have now performed a *post-hoc* comparison of participants with vs. those without previous experience in controlled breathing and included the data as a new Figure 7 (pg. 15). Participants with previous experience in controlled breathing had reduced objective breathing metrics compared to those without experience at training session 1. However, both groups of participants had a reduction in breathing rate and variability after training (pre vs. post). Despite this pilot study not being statistically powered for comparisons of users based on previous experience, this dataset suggests that Performance Edge VR can improve objective breathing metrics for both novices and trainees with experience in controlled breathing. As noted, no details were collected in the current study about the relative levels of pre-existing user experience, and this would be useful to capture in future research (pg. 16).**

*The data in figure 3 should somehow show deviation. Given the small sample size, it would be easy to show all results. Any other form of showing at least the range would be good.*

**Figures 3 has been updated to include errors bars, which has now been noted in the figure legend.**

*Statistics for figures 3 and 5 would help.*

**We believe the reviewer is referring to Figures 3 & 4. No statistical analysis was performed or reported for these datasets, as comparisons of self-reported data was not considered relevant study outcomes and the study was not powered to assess these aspects.**

*I feel the manuscript could be shorter without losing its message.*

**Efforts have been made to streamline the manuscript, while maintaining the key points and messages. Aspects of the Methods and Results relating to project scoping, approvals and initial design decisions were significantly shortened, and the overall revised manuscript length is reduced despite addition of content to address reviewers’ comments as outlined above (previous submission = 7268 words in main text; updated version = 7029 words)**
